# Peritoneal melanomatosis due to primary anorectal melanoma

**DOI:** 10.1515/pp-2025-0002

**Published:** 2025-03-26

**Authors:** Aras Emre Canda, Semra Demirli Atici, Evren Kadioglu, Cem Terzi

**Affiliations:** Surgery, KRC Clinic for Colorectal Surgery and Peritoneal Carcinomatosis, Izmir, Türkiye; Surgery, Acibadem Kent Hospital, Izmir, Türkiye; Pathology, Acibadem Kent Hospital, Izmir, Türkiye

**Keywords:** melanoma, melanomatosis, peritoneal metastasis, anorectal

Primary malignant melanoma of the anorectal region is a rare and aggressive cancer, accounting about 1 % of anorectal malignancies [1]. It is often diagnosed at advanced stages due to rapid progression and systemic metastases, resulting in poor prognosis.

Our patient presented with rectal bleeding due to primary anorectal melanoma, which had metastasized to the lungs. Full-thickness excision of the lesion in two pieces using the transanal minimally invasive surgery (TAMIS) technique confirmed the diagnosis, and systemic therapy was initiated. However, within three months, a progressive recurrent lesion in the anal canal led to obstruction, necessitating a laparoscopic diverting colostomy. During laparoscopic colostomy, extensive intraoperative melanomatosis was observed, characterized by widespread black-pigmented peritoneal nodules (Image and Supplementary Video). This finding underscores the aggressive nature of anorectal melanoma and its rapid dissemination.

Treatment options for anorectal melanoma include chemotherapy, immunotherapy, radiotherapy, and surgical resection. While cytoreductive surgery combined with hyperthermic intraperitoneal chemotherapy (HIPEC) has been reported in a single case with promising results [2], its role in the treatment of anorectal melanoma remains exploratory. Given the aggressive nature of the disease, outcomes remain frequently unfavorable due to early metastatic spread and rapid progression. Further studies are needed to evaluate the potential efficacy and safety of HIPEC in this context. Anorectal melanoma should also be considered in the differential diagnosis of benign-appearing anorectal disorders, such as thrombosed hemorrhoids, to ensure timely and accurate diagnosis.

**Figure:**
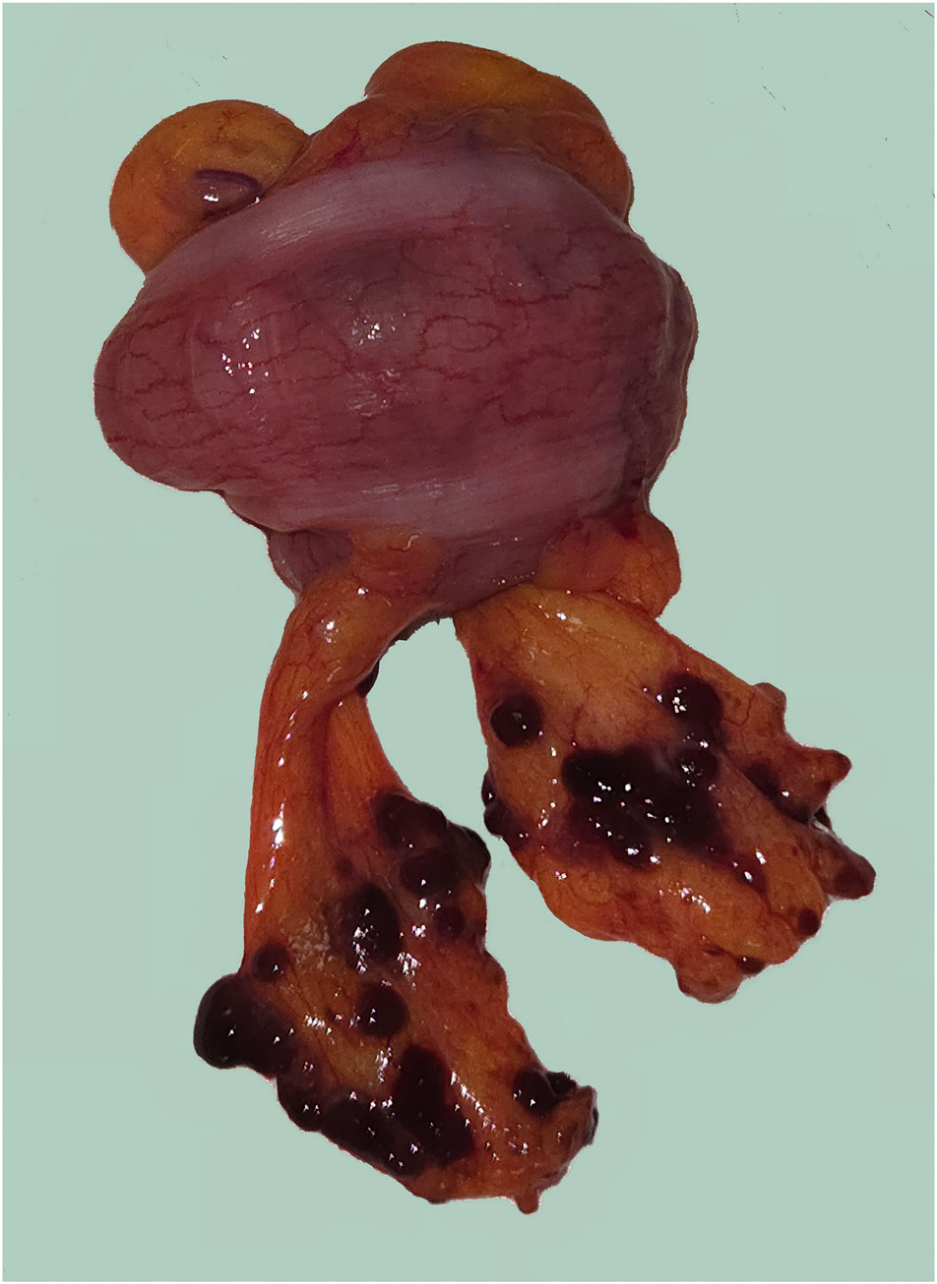


**Image:** Brownish, friable metastatic nodules measuring up to 3–5 mm on the appendices epiploicae surrounding the sigmoid colon, observed during preparation for colostomy. These nodules suggest metastatic involvement, confirming the presence of peritoneal metastases.

## Supplementary Video

Laparoscopic colostomy procedure demonstrating intraoperative melanomatosis. The video shows widespread peritoneal involvement, with multiple characteristic black-pigmented lesions.

## References

[1] Atici AE, Ozer I, Bostanci EB, Akoglu M (2009). Surgical treatment of anorectal melanoma. Turk J Colorectal Dis.

[2] Hayes-Jordan A, Green H, Prieto V, Wolff JE (2012). Unusual cases: melanomatosis and nephroblastomatosis treated with hyperthermic intraperitoneal chemotherapy. J Pediatr Surg.

